# Inflammatory and neuroendocrine biomarkers associated with mental health symptoms across body mass index categories: a cross-sectional machine learning analysis

**DOI:** 10.3389/fimmu.2026.1800205

**Published:** 2026-04-10

**Authors:** Joice M. A. Rodolpho, Krissia F. Godoy, Bruna D. L. Fragelli, Jaqueline Bianchi, Fernanda O. Duarte, Luciana Camillo, Gustavo B. Silva, Paulo Henrique Marques de Andrade, Juliana A. Prado, Evelyn Tenan Ribeiro, Francisco José Nardi Filho, Mateus Pereira Alves, Cynthia Aparecida Castro, Carlos Speglich, Fernanda F. Anibal

**Affiliations:** 1 Laboratory of Inflammation and Infectious Diseases, Federal University of São Carlos (UFSCar), São Carlos, SP, Brazil; 2 Polytechnic School - Data Science and Artificial Intelligence, Pontifical Catholic University of Campinas (PUC Campinas), Campinas, SP, Brazil; 3 Department of Medicine, Federal University of São Carlos (UFSCar), São Carlos, SP, Brazil; 4 Laboratory of Applied Math, Institute of Mathematics and Statistics - University of São Paulo (USP), Sao Paulo, SP, Brazil; 5 Laboratory of Information Systems, Institute of Computing - University of Campinas (UNICAMP), Campinas, SP, Brazil; 6 Pathology and Biocompatibility Laboratory, Federal University of São Carlos (UFSCar), São Carlos, SP, Brazil; 7 Leopoldo Américo Miguez de Mello Research Center CENPES/Petrobrás, Rio de Janeiro, Brazil

**Keywords:** artificial intelligence, depression, inflammatory biomarkers, mental health, neuroinflammation, obesity, randomForest, vitamin D

## Abstract

**Introduction:**

Obesity is increasingly recognized as a systemic condition with neuroinflammatory and psychobiological implications, yet its integrated relationship with inflammatory and neuroendocrine pathways in mental health remains insufficiently characterized. We investigated the associations between obesity, circulating biomarkers, and symptoms of depression, anxiety, stress, and post-traumatic stress disorder (PTSD).

**Methods:**

In this cross-sectional study, 251 adults were stratified according to psychiatric diagnosis and body mass index (BMI). Inflammatory, neuroendocrine, and metabolic biomarkers were quantified, including cytokines, hs-CRP, cortisol, and vitamin D. Random Forest–based machine learning models were applied to identify the relative importance of biomarkers in predicting psychological symptom severity across clinical groups and BMI categories.

**Results:**

Vitamin D, IL-6, TNF-α, and hs-CRP consistently emerged as the most relevant predictors. Individuals with severe obesity but without a formal psychiatric diagnosis exhibited an inflammatory profile comparable to that observed in patients with mental disorders, suggesting the presence of a biomarker profile potentially associated with subclinical psychobiological alterations. In participants with mental disorders, the interaction between BMI and biomarkers was more complex and widespread, indicating a state of systemic inflammatory and neuroendocrine dysregulation.

**Discussion:**

These findings indicate that extremes of nutritional status were associated with a higher frequency of inflammatory and hormonal alterations related to mental health symptoms. The integration of biomarker profiling with machine learning approaches may support early identifying patterns of biomarker relevance associated with mental health symptoms.

## 1 Introduction

Obesity has emerged as one of the most pressing global health challenges, not only because of its rapidly increasing prevalence but also due to its broad impact on morbidity, mortality, and health systems worldwide (1, 2). Beyond its well-established associations with cardiometabolic diseases, obesity is increasingly recognized as a systemic condition that affects multiple biological axes, including immune, endocrine, and neuropsychological pathways (3). Accumulating evidence indicates that individuals with obesity experience substantial impairments in quality of life, functional capacity, and psychosocial well-being, with higher rates of depression, anxiety, and stress-related disorders (4).

At the biological level, visceral adipose tissue acts as an active endocrine and immunological organ, releasing pro-inflammatory cytokines and modulating hormonal signaling, thereby sustaining a state of chronic low-grade systemic inflammation (5). This inflammatory milieu is characterized by elevated circulating levels of cytokines such as tumor necrosis factor-α (TNF-α), interleukin-6 (IL-6), and interleukin-1β (IL-1β), as well as by increased C-reactive protein (CRP), and has been consistently implicated in the pathophysiology of both metabolic and neuropsychiatric disorders (6–8). In parallel, neuroendocrine alterations involving cortisol and vitamin D have been shown to further modulate immune responses and brain functions related to mood regulation and stress reactivity (9, 10).

The relationship between obesity and mental health disorders is increasingly understood as bidirectional and biologically mediated (11–13). Chronic psychological stress may promote dysregulation of the hypothalamic pituitary adrenal (HPA) axis, favoring visceral fat accumulation, while excess adiposity amplifies systemic inflammation and neuroendocrine imbalance, potentially increasing vulnerability to depression and anxiety (14). In addition, altered cytokine levels stimulate CRP release and interfere with metabolic and neuroimmune pathways such as the kynurenine pathway, which plays a central role in tryptophan metabolism and has been implicated in the pathophysiology of depression (15, 16).

Despite growing recognition of these shared pathways, most clinical studies continue to examine inflammatory or neuroendocrine biomarkers in isolation, often using linear and univariate analytical approaches that fail to capture the complex, nonlinear, and interdependent nature of psychmetabolic regulation (17). As a result, the field still lacks robust integrative models capable of identifying biomarker patterns that reflect individual vulnerability profiles across different body mass index (BMI) categories and mental health statuses.

In this context, machine learning approaches offer a powerful framework to address the multidimensional structure of biomedical data. Among these, Random Forest (RF) algorithms are particularly well suited to clinical datasets because they can handle nonlinear interactions, correlated predictors, and imbalanced classes, while also providing interpretable measures of variable importance and internal validation through out-of-bag error estimation (18, 19). In addition, RF enables robust performance assessment using metrics such as accuracy, sensitivity, specificity, and the area under the receiver operating characteristic curve (AUC-ROC) (20).

Rather than replacing clinical reasoning, such models can serve as tools to uncover hidden biological patterns and to support identification of biomarker patterns associated with mental health symptoms in complex, multifactorial conditions (21–23) An improved understanding of how inflammatory and neuroendocrine biomarkers jointly relate to mental health symptoms across the spectrum of BMI may have important clinical implications, particularly for the identification of individuals without formal psychiatric diagnoses but presenting biological profiles that may be associated with increased psychometabolic risk.

Despite growing evidence linking obesity, inflammation, and mental health, most studies have examined biomarkers individually or within restricted clinical groups. Few investigations have integrated inflammatory, neuroendocrine, and behavioral markers across different BMI categories while simultaneously applying machine learning approaches to identify multivariate biomarker patterns associated with mental health symptoms in real-world clinical populations. Therefore, there remains a need for integrative analytical frameworks capable of exploring these complex relationships in heterogeneous clinical samples.

Therefore, the aim of this study was to investigate the integrated associations between obesity, inflammatory and neuroendocrine biomarkers, and mental health symptoms across BMI categories using a machine learning framework to identify multivariate biomarker relevance patterns in a clinically characterized sample.

## 2 Materials and methods

### 2.1 Study design and ethics

This was a cross-sectional observational study conducted in adults recruited in São Paulo State, Brazil. All participants provided written informed consent. The study protocol was approved by the Research Ethics Committee of the Federal University of São Carlos (UFSCar) and registered at Plataforma Brazil (process number: 54381621.0.0000.5504), in accordance with national and international ethical standards for research involving human subjects.

### 2.2 Participants and eligibility

. A total of 251 adults aged ≥18 years were included. Of these, 140 individuals composed the control group and 111 composed the mental health group. Participants in the mental health group had a documented psychiatric diagnosis according to the 10th revision of the International Classification of Diseases (ICD-10), based on medical records. The control group consisted of individuals without psychiatric diagnoses or related comorbidities.

Exclusion criteria for the mental health group included the presence of cardiovascular disease, autoimmune disorders, inflammatory diseases, chronic systemic diseases, or diabetes. Inclusion and exclusion criteria were applied to ensure clinical homogeneity within groups.

Participants were recruited by convenience sampling from psychiatric hospitals, Psychosocial Care Centers (CAPS), and the Federal University of São Carlos (UFSCar), São Paulo, Brazil.

### 2.3 Clinical, behavioral, and anthropometric assessment

All participants completed a standardized questionnaire (covering sociodemographic data, health status, and behavioral factors. The following variables were recorded: sex, age, weight, height, body mass index (BMI), sleep quality, smoking status, alcohol consumption, physical activity, medication use, and psychiatric diagnosis.

BMI was calculated as weight (kg) divided by height squared (m²) and categorized as: underweight (<18.5), normal weight (18.5–24.99), overweight (25.0–29.99), obesity class I (30.0–34.99), and severe obesity, including classes II (35.0–39.99) and III (≥40.0).

Sleep quality was self-reported and categorized as very good, good, poor, or very poor. Smoking status was classified as non-smoker, former smoker (abstinent for >5 years), or current smoker. Alcohol consumption was categorized as non/occasional (up to once per week) or regular (more than once per week). Physical activity was recorded as active or inactive. Medication use was recorded as yes or no.

### 2.4 Psychological assessment

Symptoms of depression, anxiety, and stress were assessed using the Depression, Anxiety and Stress Scale (DASS-21), validated for the Brazilian population. The instrument consists of 21 items, with 7 items per subscale, scored on a 4-point Likert scale ranging from 0 to 3, reflecting symptom severity over the previous week.

Scores were calculated by summing the corresponding items for each subscale and categorized according to established cutoffs. Depression was classified as normal (0–9), mild (10–13), moderate (14–20), or severe (>21). Anxiety was classified as normal (0–7), mild (8–9), moderate (10–14), or severe (≥15). Stress was classified as normal (0–14), mild (15–18), moderate (19–25), or severe (≥26).

### 2.5 Blood collection and biomarker measurements

A single venous blood sample was collected from each participant between 07:00 and 10:00 am, without prior fasting. Blood samples were collected without prior fasting because the study focused primarily on inflammatory and neuroendocrine biomarkers rather than fasting metabolic parameters. Serum was obtained by centrifugation at 4000 g for 10 minutes.

Rapid immunochromatographic assays (QuickSTAR *In Vitro*^®^) were used for the measurement of D-dimer, N-terminal pro–B-type natriuretic peptide (NT-proBNP), cortisol, vitamin D, myoglobin, troponin I, creatine kinase-MB (CK-MB), C-reactive protein (CRP), and high-sensitivity C-reactive protein (hs-CRP). Inflammatory cytokines (IL-6, IL-1β, TNF-α, and IL-8) were measured in serum using chemiluminescence (Immulite 1000).

The results for the biomarkers (**Supplementary Figure 1**) were classified as “altered” when the values ​​fell outside the reference ranges provided by the manufacturer of each assay. For rapid immunochromatographic tests (QuickSTAR *In Vitro*^®^), interpretation followed the manufacturer’s instructions and the clinical laboratory reference ranges. For cytokines measured by chemiluminescence (Immulite 1000), results were interpreted according to the standard reference ranges established by the assay manufacturer and commonly used in clinical laboratories. These categorizations were used to facilitate descriptive analyses of the frequencies of biomarker alterations in behavioral and clinical subgroups.

### 2.6 Statistical analysis

Continuous variables are presented as mean ± standard deviation (SD), and categorical variables as counts (percentages). Group comparisons were performed using the student’s t-test or Mann–Whitney U test, as appropriate, for continuous variables, and the chi-square test or Fisher’s exact test for categorical variables.

A two-sided p-value < 0.05 was considered statistically significant. Statistical analyses were conducted using GraphPad Prism version 9.0 (GraphPad Software, San Diego, CA, USA). Prior to analysis, the dataset was inspected for missing or inconsistent values and for plausibility of variable ranges. Because the analyses were primarily exploratory, p-values were not adjusted for multiple comparisons and should therefore be interpreted cautiously.

### 2.7 Machine learning analysis

To investigate the multivariate relationships between biomarkers and psychological symptom severity, we applied a machine learning framework based on Random Forest (RF). The primary objective of this analysis was not individual-level clinical prediction, but the identification and ranking of biomarkers according to their relative importance in discriminating different levels of symptom severity for depression, anxiety, and stress.

Separate RF models were constructed for each psychological outcome (depression, anxiety, and stress), using the corresponding severity categories derived from the DASS-21 as target variables. Biomarkers were used as input features.

Models were implemented using the RandomForestClassifier (n_estimators = 100, random_state = 42). Model performance was evaluated using 5-fold cross-validation, in which the dataset was randomly partitioned into five subsets; in each iteration, four subsets were used for training and one for testing. Mean accuracy across folds was used to ensure internal consistency of the models, although predictive performance itself was not the primary endpoint of the analysis.

To assess the robustness of the findings, additional ensemble classifiers (ExtraTreesClassifier and GradientBoostingClassifier) were also evaluated using the same cross-validation scheme. Because the primary objective of this study was exploratory biomarker relevance ranking rather than predictive model optimization, we focused on ensemble tree-based methods that provide interpretable feature importance measures. Performance comparisons between models were interpreted descriptively, given the exploration objective of the machine learning analysis and the absence of formal hypothesis testing for predictive performance. After model comparison, the best-performing RF model for each outcome was fitted to the full dataset to obtain measures of variable importance (feature_importances), which were used to rank biomarkers according to their relative contribution to the discrimination of symptom severity levels.

The machine learning models were used primarily as exploratory tools to evaluate the relative importance of biomarkers rather than to develop a predictive clinical model.

### 2.8 Multivariate and regression analyses

To complement the machine learning analyses, we applied two classical statistical approaches. First, multivariate analysis of variance (MANOVA) was used to assess the joint effects of independent variables on depression, anxiety, and stress scores simultaneously. Second, multiple linear regression models were fitted separately for each psychological outcome to estimate regression coefficients and assess the independent contribution of each predictor.

A significance level of p < 0.05 was adopted for all analyses.

Multivariate Analysis of Variance and Multiple linear regressions.

## 3 Results

A total of 251 participants were included in the study, comprising 140 individuals in the control group and 111 in the mental health group, classified according to ICD-10 psychiatric diagnoses. The analyses presented below should be interpreted as exploratory evaluations of associations between biomarkers, BMI categories, behavioral factors, and mental health symptoms. The sociodemographic and clinical characteristics of the sample are summarized in **Table 1**.

**Table 1:** Sociodemographic and clinical characteristics of the study population (N = 251).

Variables		Control N=140	Mental health N= 111	Value of p
**Age, Years ±SD**		39 ± 2.0	31 ± 1.81	* 0.0007
**Weight (Kg) ±SD**		74 ± 2.63	70 ± 2.56	0.85
**Height (cm) ±SD**		1.65 ± 0.014	1.65 ± 0.08	0.63
**BMI (kg/m^2^)**		25.97 ± 0.84	25.95 ± 1.04	0.64
	Underweight	6 (4.28%)	4 (3.6%)	*0.057
	Ideal	48 (34.28%)	40 (36.03%)	*0.02
	Mild obesity	75 (53.56%)	57 (51.35%)	*0.02
	Severe obesity	11 (7.84%)	10 (9%)	0.76
Sleep quality				0.60
	Good	68 (48.57%)	51 (45.94%)	
	Very Good	32 (22.85%)	27 (24.32%)	
	Bad	33 (23.57%)	23 (20.72%)	
	Very Bad	7 (5%)	10 (9%)	
Smoking				*<0.0001
	Smoker	7 (5%)	31 (27.92%)	
	Non-smoker	133 (94.99%)	80 (72.06%)	
Alcohol use pattern				0.62
	Usual	34 (24.28%)	30 (27.02%)	
	Unusual	106 (75.71%)	81 (72.97%)	

Continuous data are presented as mean ±standard deviation (SD), whereas categorical data are presented as counts (percentage). Test T nonparametric and chi-square tests were used to evaluate significant statistical differences for continuous and categorical data, respectively.

*p≤0.05 for comparisons between control vs. mental health groups.

Participants in the mental health group were significantly younger than those in the control group (31 ± 1.81 vs. 39 ± 2.0 years, respectively). The distribution of BMI categories differed modestly between groups. The mental health group showed a slightly higher proportion of individuals within the normal weight range (36.03% vs. 34.28%) and a slightly lower proportion in the overweight/obesity class I category (51.35% vs. 53.56%), while the prevalence of severe obesity was similar between groups (9.0% vs. 7.84%; p = 0.76). There was a trend toward a higher proportion of underweight individuals in the control group (4.28% vs. 3.6%; p = 0.06).

Smoking was markedly more prevalent among participants with mental disorders compared with controls (27.92% vs. 5.0%; p < 0.01). In contrast, no statistically significant differences were observed between groups with respect to body weight, height, or overall BMI. Likewise, sleep quality and alcohol consumption patterns did not differ significantly between the groups.

**Table 2** summarizes the distribution of smoking status, physical inactivity, and use of psychotropic medication according to BMI categories and mental health status.

**Table 2:** Clinical characteristics according to the degree of obesity in the studied population (N = 251).

Variables		Underweight (N/%) control	Underweight (N/%) Mental health	Value of P	BMI ideal (N/%) control	BMI ideal (N/%) Mental health	Value of P		Mild obesity (N/%) control		Mild obesity (N/%) Mental health		Value of P		Severe obesity (N/%) control		Severe obesity (N/%) Mental health		Value of P		Value of P for group	
**N**		7	6		47	40			75		55				11		10					
**Age (average)**		34	39		38	31			31		38				34		39					
Smoking				1.0			•0.00001						•0.0001						0.48		*0.0001	
	**Yes**	0	0		3 (6.38%)	28 (70%)			4 (5.33%)		16 (29.09%)				0		2 (20%)					
	**No**	7 (100%)	6 (100%)		44 (93.60%)	12 (30%)			71 (94.66%)		39 (70.9%)				11 (100%)		8 (80%)					
Sedentary lifestyle				•0.02			0.37						•0.0001						1.0		0.09	
	**Yes**	2 (28.57%)	6 (100%)		29 (61.70%)	20 (50%)			47 (62.66%)		26 (47.27%)				5 (45.45%)		5 (50%)					
	**No**	5 (71.42%)	0		18 (38.29%)	20 (50%)			28 (37.33%)		29 (52.72%)				6 (54.54%)		5 (50%)					
Use of medicationpsicotrópica				•0.0005			•0.0001						•0.0001						•0.0003		*0.0001	
	**Yes**	0	6 (100%)		0	39 (97.5%)			2 (2.66%)		54 (98.18%)				1 (9.09%)		10 (100%)				
	**No**	7 (100%)	0		47 (100%)	1 (2.5%)			73 (97.33%)		1 (1.81%)				10 (90.90%)		0					

Comparison of the prevalence of smoking, physical inactivity, and use of psychotropic medication between individuals with and without mental health conditions, stratified by BMI category (underweight, normal weight, mild obesity, and severe obesity). Categorical data are presented as counts (percentages). Chi-square test was used for categorical data when expected frequencies were > 0.5, and Fisher’s exact test when < 0.5. *p≤0.05* for comparisons across all groups; •p≤0.05 for comparisons between control vs. mental health groups within each BMI category: underweight, normal weight, mild obesity, and severe obesity.

As expected, the use of psychotropic medication was markedly more frequent among participants in the mental health group across all BMI strata, with prevalences close to or exceeding 97%, whereas its use in the control group was negligible.

Smoking was substantially more prevalent in the mental health group among individuals with normal BMI (70.0% vs. 6.38% in controls) and remained higher in the mental health group across the other BMI categories. Physical inactivity also tended to be more frequent among participants in the mental health group, particularly in the underweight category (100% vs. 28.57% in controls), although subgroup sizes were small.

These stratified analyses were conducted for descriptive and exploratory purposes only, given the limited number of participants within some BMI subgroups. Therefore, these results should be interpreted with caution and viewed as indicative of distributional patterns rather than confirmatory evidence.

**Table 3** shows the distribution of depression, anxiety, and stress symptom severity according to BMI categories in the control and mental health groups.

**Table 3:** DASS-21 clinical characteristics according to the degree of obesity in the studied population (N = 251).

		Underweight (N/%) control	Underweight (N/%) Mental health	Value of P	BMI ideal (N/%) control	BMI ideal (N/%) Mental health	Value of P	Mild obesity (N/%) control	Mild obesity (N/%) Mental health	Value of P	Severe obesity (N/%) control	Severe obesity (N/%) Mental health	Value of P	Value of P for group
Depression				0.26			•0.045			0.10			0.18	*0.0001
	**No depression**	6 (85.71%)	3 (50%)		42 (89,36%)	28 (70%)		62 (82.66%)	39 (70.90%)		9 (81.81%)	5 (50%)		
	**Mild**	0	2 (33.33%)		1 (2.12%)	3 (7.5%)		3 (4%)	4 (7.27%)		1 (9.09%)	0		
	**Moderate**	1 (14.28%)	1 (16.66%)		4 (8,51%)	8 (20%)		9 (12%)	12 (21.81%)		1 (9.09%)	5 (50%)		
	**Severe**	0	0		0	1 (2.5%)		1 (1.33%)	0		0	0		
Anxiety				1.0			0.053			0.12			0.18	*0.0001
	**No anxiety**	5 (71.42%)	4 (66.66%)		40 (85,10%)	26 (65%)		61 (81.33%)	30 (54.54%)		9 (81.81%)	5 (50%)		
	**Mild**	1 (14.28%)	0		4 (8.51%)	3 (7.5%)		2 (2.66%)	3 (5.45%)		1 (9.09%)	2 (20%)		
	**Moderate**	1 (14.28%)	2 (33.33%)		1 (2.12%)	3 (7.5%)		6 (8%)	10 (18,18%)		1 (9.09%)	2 (20%)		
	**severe**	0	0		2 (4.25%)	8 (20%)		6 (8%)	12 (21.81%)		0	1 (10%)		
Stress				0.4			0.33			0.93			0.58	0.12
	**No stress**	5 (71.42%)	6 (100%)		43 (91,48%)	33 (82.5%)		67 (89.33%)	48 (87.27%)		10 (90.90%)	8 (80%)		
	**Mild**	2 (28.57%)	0		2 (4.25%)	6 (15%)		5 (6.66%)	4 (7.27%)		1 (9.09%)	2 (20%)		
	**Moderate**	0	0		2 (4.25%)	1 (2.5%)		2 (2.66%)	3 (5.45%)		0	0		
	**Severe**	0	0		0	0		1 (1.33%)	0		0	0		

Proportion of individuals with symptoms of depression, anxiety, stress, and post-traumatic stress disorder (PTSD) in the control and mental health groups. Categorical data are presented as counts (percentages). Chi-square test was used for categorical data when expected frequencies were > 0.5, and Fisher’s exact test when < 0.5. p≤0.05 for comparisons across all groups; •p≤0.05 for comparisons between control vs. mental health groups within each BMI category: underweight, normal weight, mild obesity, and severe obesity.

Across BMI strata, participants in the mental health group consistently exhibited a higher frequency of depressive and anxiety symptoms compared with controls.

Differences between groups were more pronounced in the severe obesity category. In this subgroup, half of the participants in the mental health group presented moderate depressive symptoms, whereas most individuals in the control group reported no depression (p < 0.0001). Significant differences in depression were also observed in the underweight category (p = 0.045).

A similar pattern was observed for anxiety. In the severe obesity category, the mental health group showed a markedly higher proportion of moderate and severe anxiety compared with controls (p < 0.0001). In the other BMI categories, differences between groups were smaller and did not consistently reach statistical significance.

For stress, no statistically significant differences between control and mental health groups were observed across BMI categories.

**Figure 1:**
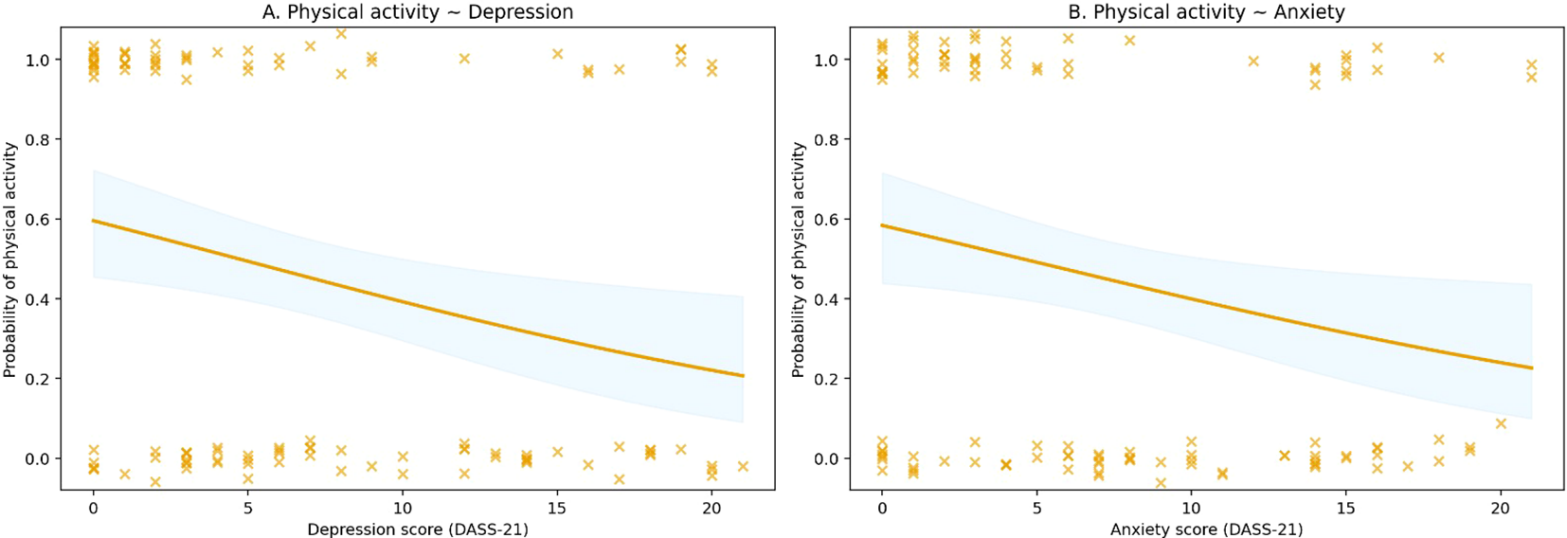
Association between physical activity and depression and anxiety symptom scores. Scatter plots show the relationship between self-reported physical activity and depression **(A)** and anxiety **(B)** symptom scores. Participants who reported regular physical activity showed lower symptom scores compared with sedentary individuals. The regression lines indicate negative associations between physical activity and symptom scores. Despite substantial inter-individual variability, the associations were statistically significant with small-to-moderate effect sizes.

In addition to categorical comparisons across groups and BMI categories, logistic regression analyses were performed to investigate associations between mental health symptom scores and the probability of engaging in physical activity. Physical activity was treated as a binary variable (physically active vs. sedentary). Physical activity emerged as the variable most consistently associated with both depression and anxiety outcomes. A significant inverse association was observed between depression scores and the probability of engaging in physical activity, such that higher depression scores were associated with a lower likelihood of being physically active (**Figure 1A**). Although substantial inter-individual variability was observed, the overall trend remained consistent, with a modest but statistically significant effect in the logistic regression model. A similar pattern was observed for anxiety, with higher anxiety scores associated with a reduced probability of engaging in physical activity (**Figure 1B****).** Because physical activity was modeled as a binary outcome, individual observations appear distributed across two levels, although the logistic regression curve illustrates the predicted probability across the range of symptom scores.

**Figure 2:**
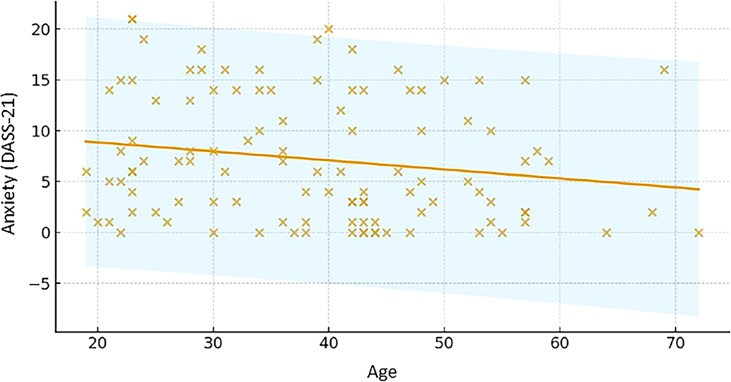
Association between age and anxiety symptom scores. Scatterplot showing the relationship between age and anxiety symptom scores. Older participants tended to report slightly lower anxiety scores, reflected by a modest but statistically significant negative association. The wide dispersion of values indicates substantial inter-individual variability, suggesting that additional factors not captured in this model may contribute to anxiety symptom severity.

Age was also independently associated with anxiety symptom scores, although with a smaller effect size. Older participants tended to report slightly lower levels of anxiety, as reflected by a modest but statistically significant negative regression coefficient (**Figure 2****).**

**Figure 3:**
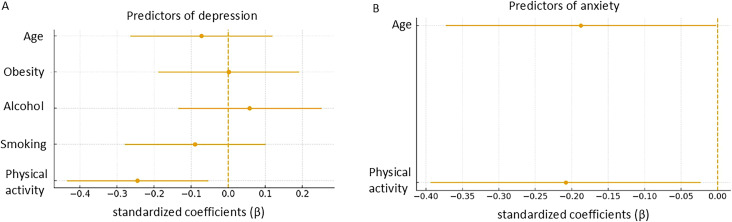
Independent associations with depression and anxiety symptom scores. Forest plots showing the regression coefficients (β) and 95% confidence intervals from the multiple linear regression models for depression **(A)** and anxiety **(B)**. Physical activity was the only variable that remained independently associated with symptom scores in the fully adjusted models, with negative β coefficients and confidence intervals not crossing zero. Other variables, including BMI category, smoking status, alcohol consumption, and age, showed coefficients close to zero and confidence intervals crossing the null line.

In the fully adjusted model for depression, physical activity was the only variable that remained independently associated with symptom scores. Other lifestyle variables, including BMI category, smoking status, alcohol consumption, and age, did not reach statistical significance, with regression coefficients close to zero and confidence intervals crossing the null line (**Figure 3A**). In contrast, in the fully adjusted model for anxiety, none of the evaluated variables showed independent associations, with all coefficients close to zero and confidence intervals crossing the null line (**Figure 3B**).

**Figure 4:**
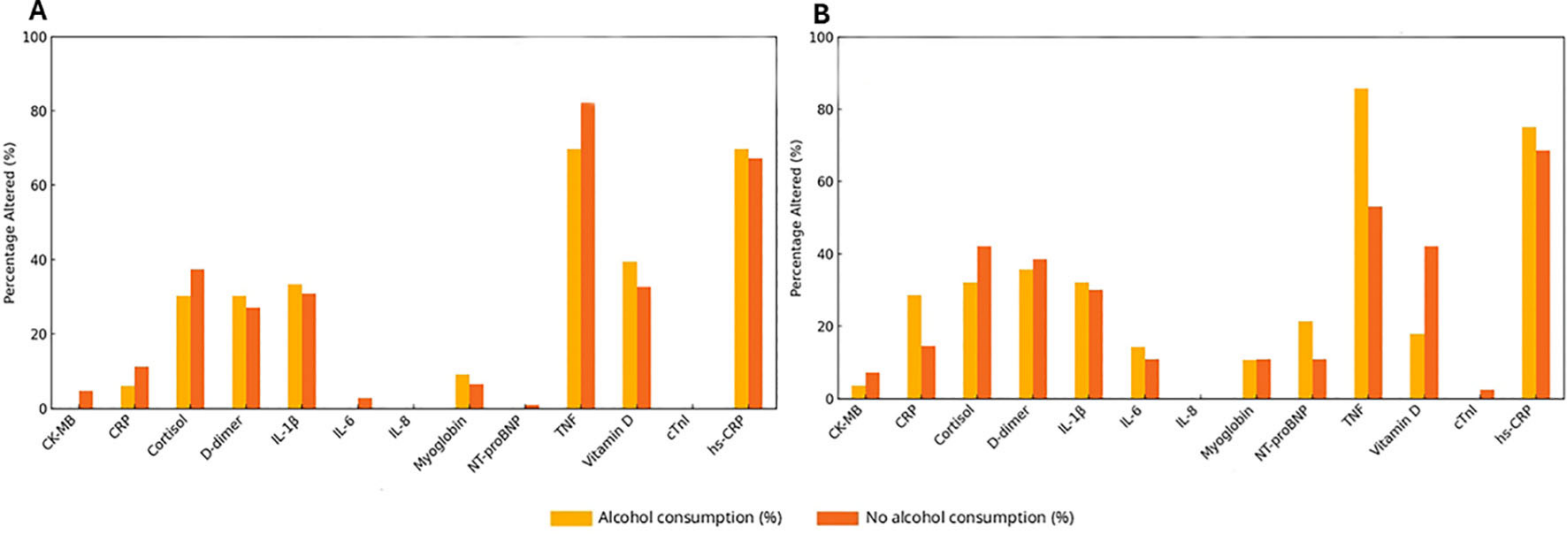
Frequency of biomarker alterations according to alcohol consumption in the control and mental health groups. Bar plots showing the percentage of individuals with altered biomarker values according to alcohol consumption status in the control group **(A)** and in the mental health group **(B)**. The biomarkers analyzed include IL-8, TNF-α, IL-1β, IL-6, vitamin D, hs-CRP (high-sensitivity C-reactive protein), CRP (C-reactive protein), NT-proBNP (N-terminal pro–B-type natriuretic peptide), myoglobin, CK-MB (creatine kinase-MB), cardiac troponin I (cTnI), and D-dimer. Values are shown as percentages for each biomarker according to alcohol consumption status. Bars indicate non/occasional drinkers and regular drinkers, as defined in the Methods.

**Figure 4** depicts the frequency of altered biomarkers according to alcohol consumption status in the control and mental health groups. In the control group (**Figure 4A**), the most frequently altered biomarkers among regular drinkers were hs-CRP (65%) and vitamin D (approximately 40%). Alterations were also observed for D-dimer and IL-1β (approximately 35%) and for myoglobin (approximately 10%), whereas other biomarkers showed low frequencies of alteration with minimal differences between regular drinkers and non/occasional drinkers.

In the mental health group (**Figure 4B****),** a higher frequency of altered biomarkers was observed among regular drinkers. TNF-α was altered in approximately 82% of regular drinkers, followed by hs-CRP (78%). Alterations were also observed for CRP and IL-1β (approximately 25%), NT-proBNP (20%), and IL-6 (approximately 15%).

Overall, regular alcohol consumption was associated with a higher frequency of biomarker alterations, particularly in the mental health group.

**Figure 5:**
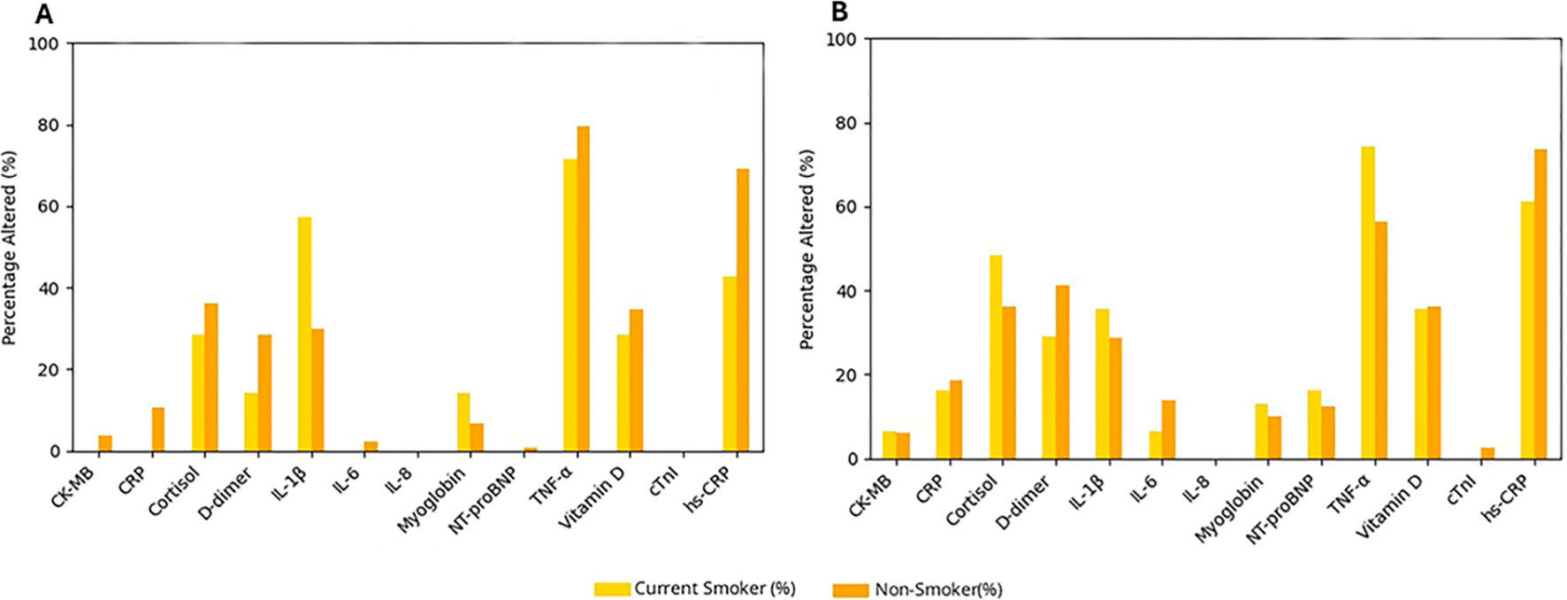
Frequency of biomarker alterations according to smoking status in the control and mental health groups. Bar plots showing the percentage of individuals with altered biomarker values according to smoking status in the control group **(A)** and in the mental health group **(B)**. The biomarkers analyzed include IL-8, TNF-α, IL-1β, IL-6, vitamin D, hs-CRP (high-sensitivity C-reactive protein), CRP (C-reactive protein), NT-proBNP (N-terminal pro–B-type natriuretic peptide), myoglobin, CK-MB (creatine kinase-MB), cardiac troponin I (cTnI), and D-dimer. Values are shown as percentages for each biomarker according to smoking status. Bars indicate smokers and non-smokers, as defined in the Methods.

**Figure 5** shows the frequency of altered biomarkers according to smoking status in the control and mental health groups. In the control group (**Figure 5A**), smokers exhibited a higher frequency of alterations in inflammatory biomarkers, particularly TNF-α (80%), followed by hs-CRP (75%), IL-6 (55%), and cortisol (35%), compared with non-smokers. Alterations were also observed in cardiovascular-related markers, including CRP (12%), D-dimer (35%), and myoglobin (15%).

In the mental health group (**Figure 5B**), a higher frequency of biomarker alterations was observed among smokers. TNF-α was altered in approximately 80% of smokers, while cortisol (approximately 55%) and IL-1β (approximately 35%) also showed elevated frequencies of alteration. In addition, alterations in cardiovascular-related biomarkers, including myoglobin and NT-proBNP (approximately 12%) and CK-MB (approximately 10%), were observed.

Overall, smoking was associated with a higher frequency of alterations across inflammatory, neuroendocrine, and cardiovascular biomarkers, particularly in the mental health group.

**Figure 6:**
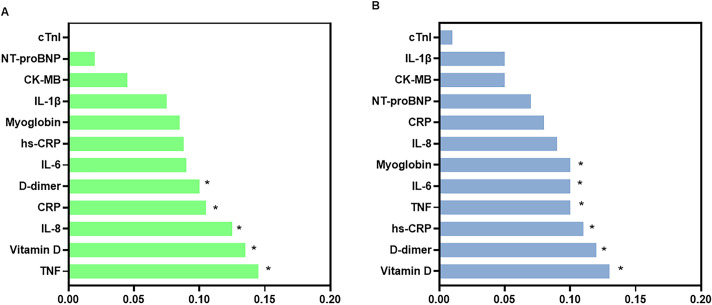
Relative importance of serum biomarkers in the Random Forest models for depression symptom severity in the control **(A)** and mental health **(B)** groups. Bar plots showing the relative importance of biomarkers in the Random Forest models used to discriminate levels of depression symptom severity in the control group **(A)** and in the mental health group **(B)**. The biomarkers analyzed include IL-8, TNF-α, IL-1β, IL-6, vitamin D, hs-CRP (high-sensitivity C-reactive protein), CRP (C-reactive protein), NT-proBNP (N-terminal pro–B-type natriuretic peptide), myoglobin, CK-MB (creatine kinase-MB), cardiac troponin I (cTnI), and D-dimer. The y-axis lists the biomarkers, and the x-axis shows their normalized relative importance in the model. Higher values indicate greater relative contribution of the variable within the model. Bars are shown separately for the control and mental health groups, as indicated in each panel.

**Figure 6** shows the relative importance of biomarkers in the Random Forest models discriminating against levels of depression symptom severity in the control and mental health groups.

In the control group (**Figure 6A**), the highest-ranked variables were TNF (importance = 0.14) and vitamin D (0.12). CRP (0.10) and D-dimer (0.097) also showed moderate importance. In contrast, cardiac-related biomarkers, including CK-MB (0.043), NT-proBNP (0.007), and troponin I, exhibited very low importance values in the model.

In the mental health group (**Figure 6B**), vitamin D remained among the highest-ranked variables (0.13), and IL-6 increased in relative importance (0.10). D-dimer (0.11), TNF (0.11), hs-CRP (0.12), and myoglobin (0.10) also ranked among the most relevant variables. As in the control group, cardiac-related biomarkers showed low importance values.

Direct comparison between groups indicated differences in the ranking of the most relevant variables: TNF showed higher relative importance in the control group, whereas IL-6 and vitamin D ranked higher in the mental health group. The relative importance of IL-8, hs-CRP, and D-dimer was similar between groups, and cardiac-related biomarkers consistently showed low importance in both models.

**Figure 7:**
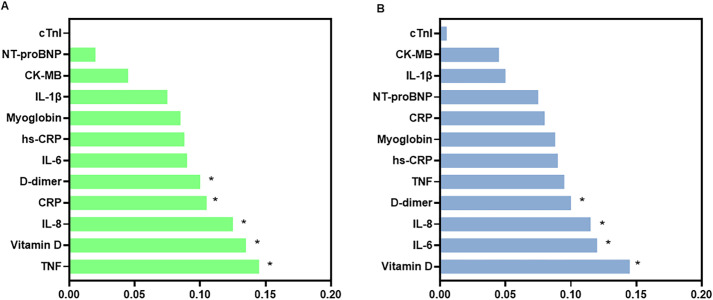
Relative importance of serum biomarkers in the Random Forest models for anxiety symptom severity in the control **(A)** and mental health **(B)** groups. Bar plots showing the relative importance of biomarkers in the Random Forest models used to discriminate levels of anxiety symptom severity in the control group **(A)** and in the mental health group **(B)**. The biomarkers analyzed include IL-8, TNF-α, IL-1β, IL-6, vitamin D, hs-CRP (high-sensitivity C-reactive protein), CRP (C-reactive protein), NT-proBNP (N-terminal pro–B-type natriuretic peptide), myoglobin, CK-MB (creatine kinase-MB), cardiac troponin I (cTnI), and D-dimer. The y-axis lists the biomarkers, and the x-axis shows their normalized relative importance in the model. Higher values indicate greater relative contribution of the variable within the model. Bars are shown separately for the control and mental health groups, as indicated in each panel.

**Figure 7** shows the relative importance of biomarkers in the Random Forest models discriminating against levels of anxiety symptom severity in the control and mental health groups.

In the control group (**Figure 7A**), the highest-ranked variables were TNF and vitamin D (both with importance = 0.14), followed by IL-8 (0.12) and CRP (0.10). In contrast, biomarkers more closely related to cardiac injury, including IL-1β, CK-MB, NT-proBNP, and troponin I, exhibited low importance values in the model.

In the mental health group (**Figure 7B****),** vitamin D again ranked among the most important variables (0.14), followed by IL-6 and IL-8. D-dimer also showed high relative importance. Myoglobin, CRP, and NT-proBNP showed intermediate importance values, whereas IL-1β, CK-MB, and troponin I ranked among the lowest.

Overall, comparison between groups indicated differences in the ranking of the most relevant biomarkers, while cardiac-related biomarkers consistently showed low importance values in both models.

**Figure 8:**
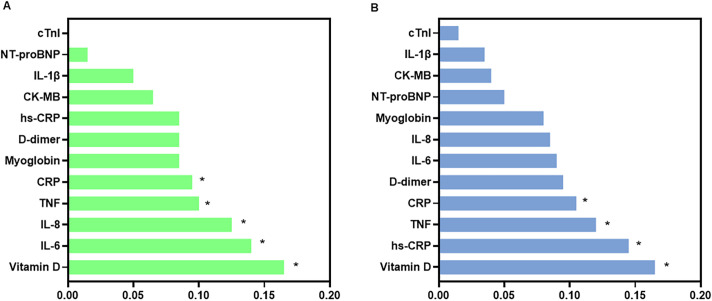
Relative importance of serum biomarkers in the Random Forest models for stress symptom severity in the control **(A)** and mental health **(B)** groups. Bar plots showing the relative importance of biomarkers in the Random Forest models used to discriminate levels of stress symptom severity in the control group **(A)** and in the mental health group **(B)**. The biomarkers analyzed include IL-8, TNF-α, IL-1β, IL-6, vitamin D, hs-CRP (high-sensitivity C-reactive protein), CRP (C-reactive protein), NT-proBNP (N-terminal pro–B-type natriuretic peptide), myoglobin, CK-MB (creatine kinase-MB), cardiac troponin I (cTnI), and D-dimer. The y-axis lists the biomarkers and the x-axis shows their normalized relative importance in the model. Higher values indicate greater relative contribution of the variable within the model. Bars are shown separately for the control and mental health groups, as indicated in each panel.

**Figure 8** shows the relative importance of biomarkers in the Random Forest models discriminating against levels of stress symptom severity in the control and mental health groups.

In the control group (**Figure 8A**), vitamin D showed the highest relative importance (0.16), followed by IL-6 (0.14), IL-8 (0.13), and TNF (0.10). CRP also ranked among the variables with moderate importance. In contrast, IL-1β, NT-proBNP, and troponin I exhibited low importance values in the model.

In the mental health group (**Figure 8B**), vitamin D is again ranked as the most important variable (0.16), followed by hs-CRP (0.12) and TNF (0.11). CRP showed intermediate importance, whereas myoglobin and NT-proBNP had lower relative importance values. CK-MB, IL-1β, and troponin I ranked among the lowest.

Overall, comparison between groups indicated differences in the ranking of the most relevant biomarkers, while cardiac-related biomarkers consistently showed low importance values in both models.

**Figure 9:**
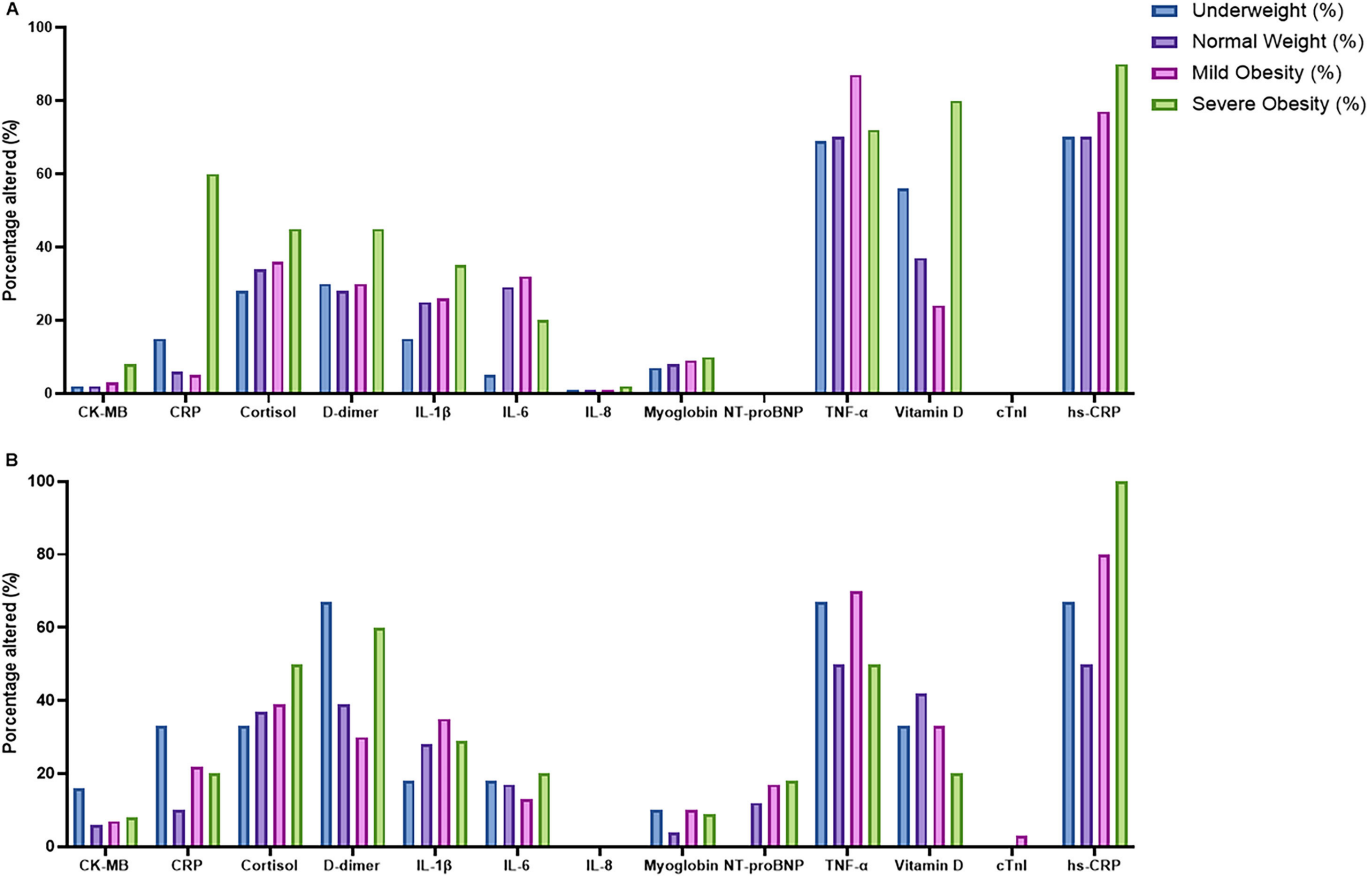
Frequency of altered biomarkers according to BMI category in the control and mental health groups. Bar plots showing the percentage of individuals with altered biomarker values across BMI categories in the control group **(A)** and in the mental health group **(B)**. The biomarkers evaluated include CK-MB, CRP, cortisol, D-dimer, IL-1β, IL-6, IL-8, myoglobin, NT-proBNP, TNF-α, vitamin D, cardiac troponin I (cTnI), and hs-CRP. Values are shown as percentages of individuals with altered results within each BMI category (underweight, normal weight, overweight/obesity class I, and severe obesity), as defined in the Methods.

**Figure 9** shows the frequency of altered biomarkers according to BMI category (BMI < 25 vs. BMI ≥ 25) in the control and mental health groups.

In the control group (**Figure 9A**), participants with BMI ≥ 25 showed a substantially higher frequency of biomarker alterations compared with those with BMI < 25. The most frequent alterations among individuals with BMI ≥ 25 were observed for TNF, D-dimer, and hs-CRP (approximately 80%), followed by cortisol and D-dimer (approximately 45%). Alterations in myoglobin and CRP were also observed (approximately 60%), whereas cardiac-related biomarkers, including NT-proBNP, CK-MB, and troponin I, remained infrequent. In contrast, participants with BMI < 25 exhibited markedly lower frequencies of altered biomarkers across all markers.

In the mental health group (**Figure 9B**), a similar but more accentuated pattern was observed. Among participants with BMI ≥ 25, hs-CRP was altered in nearly all individuals (approximately 100%). Higher frequencies of alterations were also observed for D-dimer (approximately 60%), TNF, and cortisol (approximately 45%). As in the control group, participants with BMI < 25 showed lower frequencies of altered biomarkers.

Overall, higher BMI was associated with a higher frequency of biomarker alterations in both groups, with a more pronounced pattern in the mental health group.

**Figure 10:**
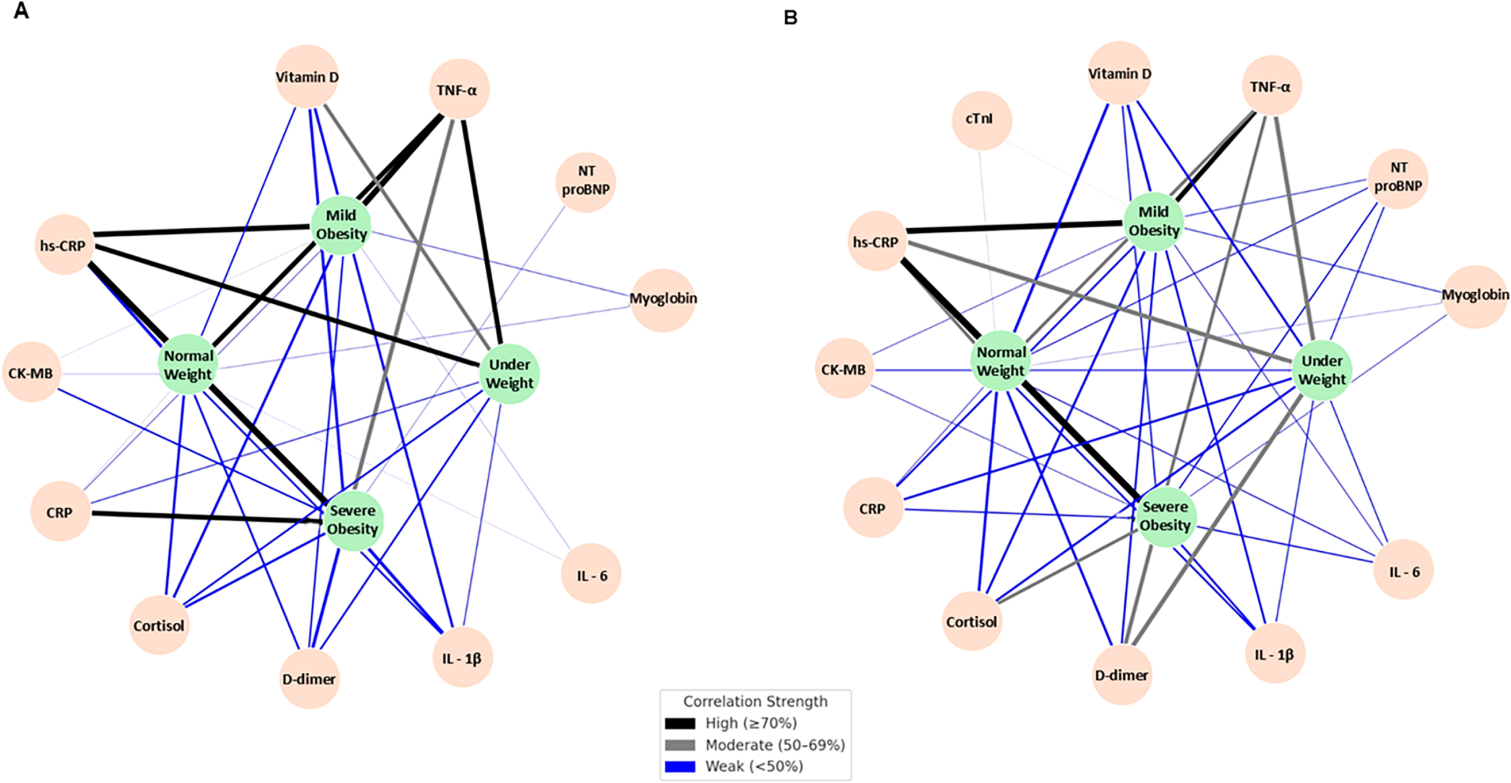
Network of associations between biomarkers and BMI categories in the control **(A)** and mental health **(B)** groups. Network graphs showing the associations between biomarkers and BMI categories in individuals without **(A)** and with **(B)** mental health disorders, based on the percentage of individuals with altered biomarker values. Nodes represent biomarkers and BMI categories. Edges indicate the strength of association according to the frequency of altered values: black lines represent strong associations (≥70%), grey lines indicate moderate associations (50–69%), and blue lines represent weak associations (<50%).

**Figure 10** shows the network of associations between biomarkers and BMI categories in the control group (**Figure 10A**) and in the mental health group (**Figure 10B**), based on the percentage of individuals with altered biomarker values. Edges represent the strength of association: black lines indicate strong associations (≥70%), grey lines indicate moderate associations (50–69%), and blue lines indicate weak associations (<50%).

In the control group (**Figure 10A**), hs-CRP and TNF-α appeared as the main nodes with the highest number of connections across BMI categories, particularly with underweight, overweight/obesity, and severe obesity, mainly through strong associations. In addition, several weak associations were observed between inflammatory and neuroendocrine biomarkers and BMI categories, including cortisol, IL-6, IL-1β, and D-dimer, especially in the severe obesity category.

In the mental health group (**Figure 10B**), a higher density of connections between biomarkers and BMI categories was observed. hs-CRP remained one of the most connected nodes, showing strong associations with normal weight, overweight/obesity, and severe obesity. TNF-α also showed multiple moderate-to-strong associations, particularly with underweight and overweight/obesity. In addition, a larger number of moderate and weak associations involving IL-6, IL-1β, cortisol, and D-dimer were observed, especially in the severe obesity category.

Overall, compared with the control group, the mental health group exhibited a denser and more distributed network of associations, characterized by a greater number of moderate and weak connections involving multiple biomarkers across BMI categories.

## 4 Discussion

In this study, we investigated the integrated relationships between obesity, mental disorders, and inflammatory and neuroendocrine biomarkers in a sample of 251 individuals, using Random Forest (RF) as an analytical tool. Overall, the results indicate a consistent pattern of greater biomarker alterations among individuals with mental disorders, particularly at the extremes of BMI, consistent with the concept of a potential interface between obesity, systemic inflammation, and psychological distress (4, 24).

A central observation was the higher frequency of alterations in inflammatory biomarkers such as IL-6, TNF-α, hs-CRP, and D-dimer among individuals with psychiatric diagnoses, especially those with severe obesity. In this subgroup, alterations in hs-CRP exceeded 90% and vitamin D alterations were also highly prevalent. This pattern is in line with the extensive literature describing obesity as a state of chronic low-grade inflammation and consistent with previous studies reporting associations between obesity, systemic inflammation, and mood and anxiety disorders (25, 26).

Across all symptom domains—depression, anxiety, and stress—vitamin D consistently ranked among the most relevant biomarkers in the RF models in both groups. This finding is consistent with previous studies reporting associations between vitamin D deficiency and mood disorders, particularly in overweight and obese populations, and supports its recognized immunomodulatory and neurobiological relevance (27).

In addition to biological markers, lifestyle-related variables also showed meaningful associations with mental health outcomes in the classical regression analyses. Physical activity was independently associated with lower depression and anxiety scores, whereas age showed a modest inverse association with anxiety symptoms. These results are consistent with a growing body of evidence indicating that behavioral factors are associated with biological and psychological profiles related to mental health outcomes (28, 29).

Although causal inference cannot be drawn from the present cross-sectional design, these findings align with previous studies reporting beneficial associations of physical activity with mental health.

Several mechanistic pathways have been proposed to explain these associations. Regular physical activity has been associated with lower circulating levels of pro-inflammatory cytokines, including IL-6 and TNF-α, and with higher levels of anti-inflammatory mediators (30, 31), potentially mitigating neuroinflammatory processes implicated in depression and anxiety (4). In addition, exercise has been linked to modulation of the hypothalamic–pituitary–adrenal (HPA) axis and to changes in neuroplasticity-related pathways, including brain-derived neurotrophic factor (BDNF) signaling (32, 33). The modest inverse association between age and anxiety observed here is also consistent with epidemiological data indicating higher prevalence of anxiety disorders in younger adults, with a tendency to decline with age (34).

Behavioral factors such as smoking and alcohol consumption were also associated with a higher frequency of biomarker alterations, particularly in the mental health group. For instance, smokers with mental disorders showed high frequencies of alterations in TNF-α, IL-1β, and cortisol. These observations agree with previous studies suggesting that smoking and alcohol use are associated with higher inflammatory burden and neuroendocrine dysregulation, especially in psychiatric populations (35).

The application of Random Forest in this context allowed not only the evaluation of model performance, but more importantly the identification of biomarkers with higher relative importance in discriminating levels of symptom severity. Across depression, anxiety, and stress models, inflammatory markers (including IL-6, IL-8, TNF, and hs-CRP) and neuroendocrine markers (particularly vitamin D and cortisol) consistently ranked higher than classical cardiac injury markers such as CK-MB, NT-proBNP, and troponin I. This pattern suggests that, within this sample, the immuno-neuroendocrine axis is more closely associated with mental symptom severity than overt cardiovascular injury markers.

This observation is consistent with current models proposing that psychiatric disorders have been reported to share inflammatory and neuroendocrine pathways with metabolic conditions (36, 37). Chronic activation of this axis and sustained low-grade inflammation may jointly contribute to alterations in brain regions involved in emotional regulation, such as the hippocampus and prefrontal cortex (36, 37). Vitamin D deficiency, which has also been linked to mood disorders, may further interact with these pathways through effects on immune regulation and neuroendocrine signaling.

The network analysis further supported a more complex and denser pattern of associations between BMI categories and biomarkers in individuals with mental disorders compared with controls. Although these analyses are descriptive, they are consistent with the notion that psychiatric conditions are accompanied by a broader and more distributed pattern of biological alterations, as reported in studies describing neuroinflammation and HPA axis dysfunction in mood disorders (36, 38).

While BMI remains a widely used and practical metric in both clinical and epidemiological settings, its limitations in capturing the metabolic and inflammatory heterogeneity of obesity are increasingly recognized, particularly in the presence of psychiatric comorbidities (39–42). Recent studies suggest that BMI should be interpreted alongside clinical and biochemical markers to better reflect cardiometabolic and neuropsychiatric risk (43, 44). In this context, the present findings support the value of integrating anthropometric measures with inflammatory and neuroendocrine biomarkers to achieve a more nuanced characterization of psych metabolic risk.

Methodologically, this study contributes by applying a machine learning approach in a clinically stratified sample, enabling the integration of multiple interrelated biomarkers and clinical variables. Random Forest proved useful for handling correlated predictors and for ranking variables according to their relative importance, an approach that may be informative in future hypothesis-driven and longitudinal studies (45, 46). Several limitations should be acknowledged. The cross-sectional design precludes any causal inference; therefore, the findings should be interpreted as exploratory associations rather than causal relationships. In addition, lifestyle and clinical factors such as smoking and the use of psychotropic medications differ substantially between groups and may influence inflammatory and neuroendocrine biomarkers. The sample was recruited by convenience from a single region, which may limit the generalizability of the findings. Furthermore, although the machine learning analyses were internally validated, no external validation cohort was available. In addition, because the machine learning models were used primarily for exploratory biomarker relevance ranking, the analysis did not focus on individual prediction errors or detailed failure case analysis. These aspects should be addressed in future perspective and multicenter studies. An additional limitation relates to the clinical heterogeneity of the psychiatric group. Participants were classified according to ICD-10 diagnoses documented in medical records, encompassing a range of psychiatric conditions. Although this approach reflects real-world clinical populations, it may mask disorder-specific biomarker patterns that could differ across diagnostic categories. The present study was therefore designed from a transdiagnostic perspective, focusing on dimensional mental health symptoms assessed by DASS-21 rather than diagnosis-specific biological signatures. This approach is consistent with contemporary frameworks in psychiatric research, such as the Research Domain Criteria (RDoC), which emphasize dimensional symptom domains and shared biological mechanisms across traditional diagnostic categories. Future studies with larger samples may allow stratified analyses exploring biomarker profiles within more homogeneous diagnostic subgroups.

In summary, the present findings support the relevance of an integrated psych metabolic perspective, in which inflammatory, neuroendocrine, and behavioral factors are considered jointly in relation to mental health symptoms. The convergence of obesity, systemic inflammation, and psychological distress emerges as a clinically meaningful pattern, and the combined use of biomarker profiling and multivariate analytical approaches may help refine risk stratification and guide future research in precision psychiatry and psych metabolic medicine.

## 5 Conclusions

This study identifies consistent associations between mental disorders, systemic inflammation, and nutritional status. Individuals with psychiatric diagnoses showed a higher frequency of alterations in inflammatory biomarkers, including IL-6, TNF, hs-CRP, and IL-8, indicating a closer link between mental health symptoms and low-grade inflammatory activity. The machine learning analyses identified a set of inflammatory and neuroendocrine biomarkers that ranked consistently among the most relevant variables for discriminating levels of depression, anxiety, and stress symptom severity.

In addition, the network analysis revealed a denser and more complex pattern of associations between biomarkers and BMI categories in individuals with mental disorders compared with controls, suggesting a broader and more distributed biological signature in this group. Together, these findings highlight the value of integrating anthropometric, inflammatory, and neuroendocrine markers in the study of psych metabolic vulnerability and support the use of multivariate analytical approaches to better characterize the biological heterogeneity underlying mental health symptoms.

## Data Availability

The original contributions presented in the study are included in the article/**Supplementary Material**. Further inquiries can be directed to the corresponding authors.
