# Supplementary material for: Inflammatory and neuroendocrine biomarkers associated with mental health symptoms across body mass index categories: a cross-sectional machine learning analysis
Source: Front Immunol. 2026 Apr 10;17:1800205. doi: 10.3389/fimmu.2026.1800205 (PMC13105972; doi:10.3389/fimmu.2026.1800205)
Supplement: Supplementary file 1 [file SupplementaryFile1.docx]

| Biomarkers | Reference values |
| --- | --- |
| D – Dimer | (0-0,5) mg/L |
| CK-MB | (0-5) ng/mL |
| cTnI | (0-0,3) ng/mL |
| Myo | (0-58) ng/mL |
| Nt-ProBNP | (0-300 <75 years  0-450 >75 years - pg/mL) |
| Cortisol | (201,31-536,54 nmol/L) |
| CRP | (0-10) mg/L |
| HsCRP | (0-1) mg/L |
| Vitamin D | >30 ng/mL |
| IL-6 | (5,9 pg/mL) |
| IL-1β | (5 pg/mL) |
| TNF | (8,1 pg/mL) |
| IL-8 | (62 pg/mL) |

**Figure S1**: Reference values biomarkers used in the study

**Legend:** Reference values for the evaluated biomarkers according to the specifications provided by the manufacturers of the diagnostic kits used in this study. The ranges include cardiovascular biomarkers (D-dimer, CK-MB, cTnI, myoglobin, and NT-proBNP), inflammatory markers (CRP, hsCRP, IL-6, IL-1β, TNF, and IL-8), cortisol, and vitamin D. These values were used as reference intervals for the interpretation of the measured concentrations.
